# Molecular Etiology of Atherogenesis – *In Vitro* Induction of Lipidosis in Macrophages with a New LDL Model

**DOI:** 10.1371/journal.pone.0034822

**Published:** 2012-04-13

**Authors:** Luis M. B. B. Estronca, Joao C. P. Silva, Julio L. Sampaio, Andrej Shevchenko, Paul Verkade, Alfin D. N. Vaz, Winchil L. C. Vaz, Otilia V. Vieira

**Affiliations:** 1 Center for Neuroscience and Cell Biology, University of Coimbra, Largo Marquês de Pombal, Coimbra, Portugal; 2 Max-Planck Institute for Molecular Cell Biology and Genetics. Pfotenhauerstrasse, Dresden, Germany; 3 Schools of Biochemistry, and Physiology and Pharmacology, Medical Sciences, University of Bristol, Bristol, United Kingdom; 4 Pharmacokinetics, Dynamics & Metabolism, Pfizer Global Research and Development, Groton, Connecticut, United States of America; 5 Department of Chemistry, University of Coimbra, Coimbra, Portugal; The University of Queensland, Australia

## Abstract

**Background:**

Atherosclerosis starts by lipid accumulation in the arterial *intima* and progresses into a chronic vascular inflammatory disease. A major atherogenic process is the formation of lipid-loaded macrophages in which a breakdown of the endolysomal pathway results in irreversible accumulation of cargo in the late endocytic compartments with a phenotype similar to several forms of lipidosis. Macrophages exposed to oxidized LDL exihibit this phenomenon *in vitro* and manifest an impaired degradation of internalized lipids and enhanced inflammatory stimulation. Identification of the specific chemical component(s) causing this phenotype has been elusive because of the chemical complexity of oxidized LDL.

**Methodology/Principal Findings:**

Lipid “core aldehydes" are formed in oxidized LDL and exist in atherosclerotic plaques. These aldehydes are slowly oxidized *in situ* and (much faster) by intracellular aldehyde oxidizing systems to cholesteryl hemiesters. We show that a single cholesteryl hemiester incorporated into native, non-oxidized LDL induces a lipidosis phenotype with subsequent cell death in macrophages. Internalization of the cholesteryl hemiester *via* the native LDL vehicle induced lipid accumulation in a time- and concentration-dependent manner in “frozen" endolysosomes. Quantitative shotgun lipidomics analysis showed that internalized lipid in cholesteryl hemiester-intoxicated cells remained largely unprocessed in those lipid-rich organelles.

**Conclusions/Significance:**

The principle elucidated with the present cholesteryl hemiester-containing native-LDL model, extended to other molecular components of oxidized LDL, will help in defining the molecular etiology and etiological hierarchy of atherogenic agents.

## Introduction

Atherogenesis is a slow progressive process characterized by a complex sequence of events. It is initiated by subendothelial retention and subsequent processing of low density lipoproteins (LDL) in the arterial *intima* leading to endothelial activation, trans-endothelial migration of monocytes, their conversion to macrophages, lipid accumulation in the macrophages and arterial smooth muscle cells, their subsequent apoptosis, and defective clearance of the apoptotic debris. A “fatty streak" that reflects deposits of lipid in the *intima* is the first visible sign of this pathology (reviewed in [1]–[5]).

Normally, LDL endocytosed by cells *via* the LDL-receptor are delivered to lysosomes where their cholesteryl esters are hydrolyzed by acid hydrolases to free cholesterol that is then either exported from the cell or re-esterified in the endoplasmic reticulum and stored in the cytosol in lipid storage droplets [1]. Modified LDL are internalized by macrophages *via* a large number of receptors that are not particularly specific to LDL [6], or even *via* macropinocytosis [7]. It is the post-internalization processing of LDL by sub-endothelial cells (including macrophages and smooth muscle cells) that determines whether the ingested material is used and/or re-cycled, or becomes toxic to these cells and elicits a sequence of signaling events and, ultimately, cell death. Cells that take up large amounts of modified LDL particles develop the microscopic appearance that has come to be known as “foam cells" [8]. However, foam cells, as they appear in light microscopy, may be of different types − one in which the rapidly internalized lipid is duly processed and stored in lipid droplets for later use, another in which the lipid accumulates irreversibly in a “frozen" endolysosomal compartment due to impairment of some activity that is vital for further processing. The latter type of lipid engorged endolysosomes, also manifested in several lipid-storage disorders, are pathological, lead to cell death, and are a hallmark of atherogenesis (reviewed in [9]).

The “oxidative-modification of LDL" hypothesis [10], one of the oldest and, arguably, most tested hypothesis for the etiology of atherogenesis, has been reviewed from several perspectives over the past 25 years [2], [9]–[17]. Oxidized LDL (Ox-LDL) exist *in vivo* in the artery wall and stimulate endothelial cells to produce pro-inflammatory molecules that recruit monocytes and promote their differentiation to macrophages. The macrophages then internalize the Ox-LDL, are unable to metabolically handle it, and begin to accumulate ingested cargo irreversibly in the endolysosomal compartment in a manner similar to that seen in lipid storage diseases [9]. Other hypotheses for the chemical etiology of atherogenesis have been proposed [3], [5], [18], but do not necessarily invalidate the idea that oxidative modifications of subendothelial-trapped LDL may be simultaneously at work.

**Figure 1:**
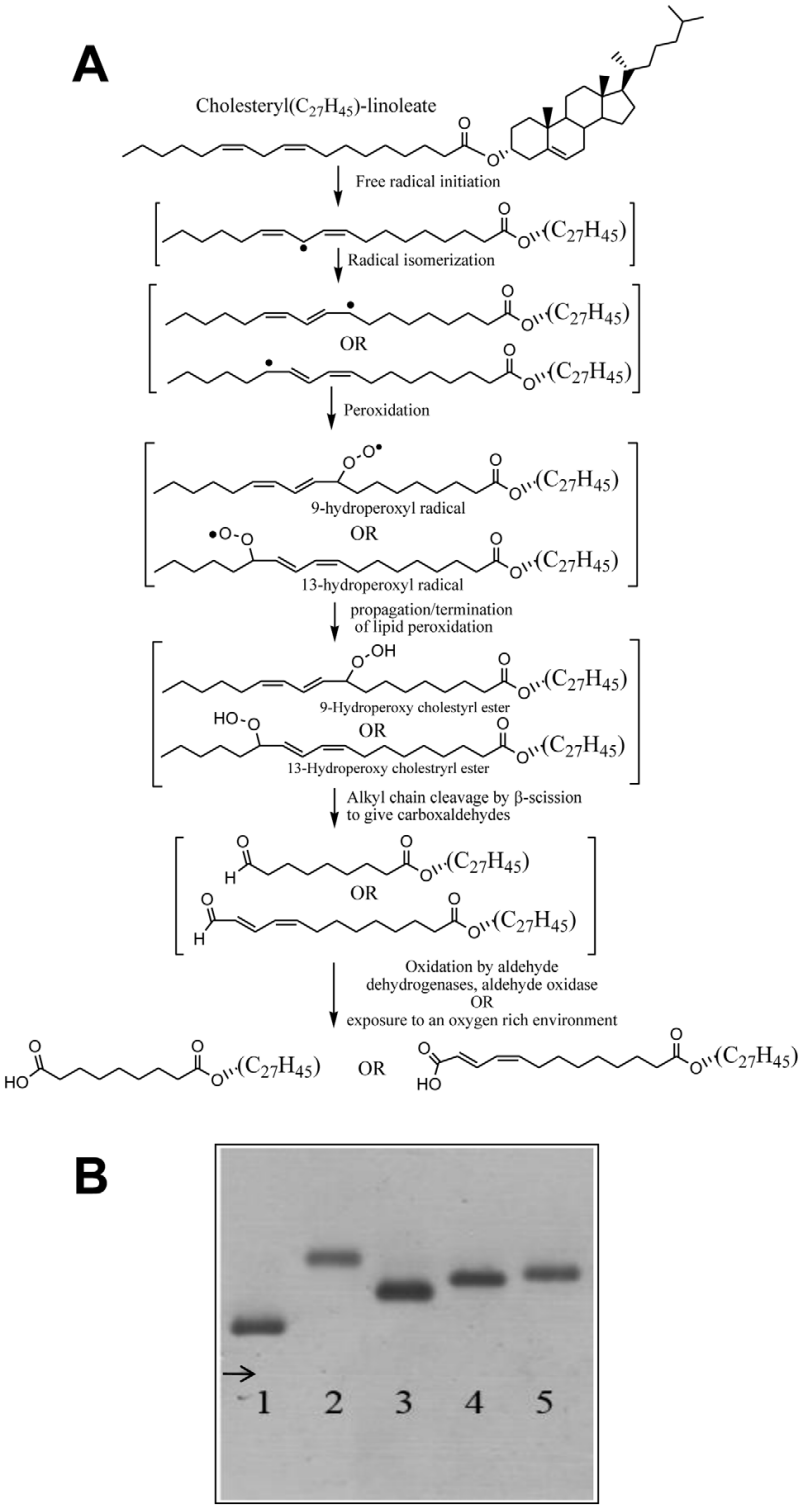
Chs increases the negative charge of Nat-LDL. (A) A brief description of the oxidation of cholesteryl linoleate to cholesteryl hemiesters; (B) Agarose gel electrophoresis of Nat-LDL, and derivatives. Lane 1, Nat-LDL; Lane 2, Ac-LDL; Lane 3, Chs-LDL (250∶1); Lane 4, Chs-LDL (500∶1); and Lane 5, Chs-LDL (1000∶1).

Ox-LDL contain a myriad of lipid oxidation products with biological activity. Cholestryl linoleate and cholesteryl arachidonate, the predominant cholesteryl esters with more than one double bond in LDL, produce several aldehydes upon oxidation: some (such as 4-hydroxynon-2-enal and malondialdehyde) are quite water-soluble while others are less polar and have variable degrees of partitioning between lipid and aqueous phases [19]. The latter group of aldehyde products most of which have cholesterol or derivatives of cholesterol as part of their structure, have come to be known as “core-aldehydes", are present in atherosclerotic lesions and resist hydrolysis upon internalization by macrophages [20], [21]. Being amphiphilic molecules, they may be expected to partition between the lipid environment in which they are formed, cell membranes, the (intra- and extracellular) aqueous phase, and translocate readily across cellular membranes. With time, in an oxidizing environment, or *via* the action of intracellular aldehyde oxidizing systems, they will be oxidized to stable cholesteryl hemiesters of the corresponding bi-acids as shown in Figure 1A. Cholesteryl-hemiesters have been identified *in vitro* as components of Ox-LDL [22], and are ligands for β_2_-glycoprotein 1, a major antigen for antiphospholipid antibodies present in patients with antiphospholipid syndrome in which high serum levels of the complexes of Ox-LDL with β_2_-glycoprotein 1 are associated with arterial thrombosis [23].

Several experimental models of Ox-LDL have been used to examine the molecular mechanisms of the phenomenology described above. The physiological relevance of methods used *in vitro* to oxidize LDL is debatable, and the complexity of the product with regard to the oxidized components makes these models of Ox-LDL difficult to work with. This is particularly applicable to comparisons of studies from different laboratories since the detailed chemical composition of Ox-LDL is almost never reported and preparations isolated from tissues vary greatly among laboratories (reviewed in [17]). The large number of potentially bioactive products of lipid peroxidation in Ox-LDL makes it practically impossible to associate a specific biological response with a specific chemical component of the Ox-LDL models. To circumvent this problem we have developed an LDL model in which native LDL (Nat-LDL) are enriched in a single chemical species that is one of the stable end products of oxidation of native cholesteryl esters. We have focused on the stable hemiesters of cholesterol that are expected to be formed from the cholesteryl ester aldehydes that result from the peroxidation of cholesteryl esters (see Figure 1A). This group of compounds has an unmodified cholesterol moiety esterified to one of the carboxylic acid groups of short chain aliphatic dicarboxylic acids (mostly azelaic acid) and, to our knowledge, their potential atherogenicity has been largely ignored in the literature. Under physiological conditions the negative charge of the cholesteryl hemiesters increases their potential to partition into the aqueous phase while the cholesteryl moiety favors their partitioning into lipid phases (including all types of cell membranes). When formed in Ox-LDL cholesteryl hemiesters may be expected to accumulate at the polar surface of the LDL particles, to which they would impart a negative surface charge, and rapidly partition *via* passive diffusion and trans-membrane translocation into all membranes and the cytosol of neighbouring cells or cells that have internalized Ox-LDL.

We began by assessing the effect of LDL charge, resulting from the incorporation of a cholesteryl hemiester, cholesteryl hemisuccinate (Chs), in Nat-LDL. Specifically, we examined the involvement of Chs in the formation of a lipidosis phenotype and in cell death when macrophages were exposed to Chs-loaded Nat-LDL (Chs-LDL). We show that Chs is sufficient to induce a lipidosis phenotype and apoptotic cell death, two characteristic features of Ox-LDL. We also show that Chs-LDL lead mainly to lipid over-accumulation in endolysosomal structures, not lipid-storage organelles. Our results suggest that *in vitro* cholesteryl hemiesters alone are able to induce lipidosis, typically seen in lipid storage pathologies and atherosclerosis, and that this process may also have relevance *in vivo*. We propose that this model can be used to systematically screen the effects of various other components of Ox-LDL and establish clear hierarchies in the molecular etiology of atherogenic lipidosis.

## Results

### Incorporation of Chs into LDL generates negatively charged particles

Several *in vitro* experimental Ox-LDL models have been used to examine the molecular etiology of atherogenic lipidosis in macrophages and, consequently, elucidate the origin of atherogenesis. However, the multitude of oxidation products in Ox-LDL makes it difficult to establish etiological hierarchies. To avoid this complication and study the biological effects of a specific family of compounds that may be formed upon oxidation of LDL, we generated a new model of LDL enriched specifically with Chs as a model cholesteryl hemiester. Cholesteryl-4-oxobutyrate, a precursor of Chs, is one of the “core aldehyde" products of the peroxidation of LDL [20]. Cholesteryl hemiesters are known to be formed *in vitro* under conditions of oxidative stress, and have been identified as ligands for the plasma protein β_2_GPI [23]. Elevated levels of Ox-LDL/β_2_GPI complexes *in vivo* seem to be correlated with acute coronary syndromes [24], [25]. Chs has all the fundamental physico-chemical properties (amphiphilicity, charge, apolar structure and potential to partition into and translocate across membranes) of the cholesteryl hemiesters expected to be formed from cholesteryl esters in LDL (Figure 1A and [20], [26]). Thus, we addressed the involvement of Chs-LDL in the induction of lipidosis and posterior death in macrophages.

Chs-LDL were generated by incubating Nat-LDL with Chs-containing POPC liposomes (with a Chs/POPC molar ratio of 70∶30) at different LDL∶liposome ratios. After incubation, the LDL particles were re-isolated by ultracentrifugation (see Methods) to eliminate unincorporated Chs and liposomes. Chs incorporation into the LDL was assessed by measuring radioactivity of the LDL particles after incubation with liposomes containing ^3^H-Chs and by assessing the LDL charge by electrophoretic mobility in an agarose gel. Figure 1B shows the electrophoretic mobility of Nat-LDL (lane 1), acetylated LDL (Ac-LDL) (lane 2), and LDL enriched in Chs at different Chs/LDL molar ratios (250∶1-lane 3; 500∶1-lane 4 and 1000∶1-lane 5). The incorporation of Chs into LDL gives these particles a more negative surface charge when compared with Nat-LDL. However, the electrophoretic mobility of Ac-LDL is higher than that of Chs-LDL even at a Chs/LDL ratio of 1000∶1. The possibility that spontaneous oxidation of LDL and consequent alterations in LDL charge might occur during Chs loading was confirmed by treating Nat-LDL similarly, but excluding Chs. These control LDL were shown to have an unchanged electrophoretic mobility. Thus, the LDL were neither significantly oxidized nor aggregated during Chs-loading.

Since this work is an attempt to present a new approach, and to establish a proof of concept, to study the molecular etiology of lipidosis *in vitro*, it must be emphasized here that Chs-LDL particles are only a model for cholesteryl hemiester-containing LDL without any other alteration of the chemical constitution of the particles. We emphasize here that cholesteryl hemiesters have been largely ignored in the literature as potential causes of atherogenesis.

### Nat-LDL enriched in Chs induce lipid accumulation and cell death

**Figure 2:**
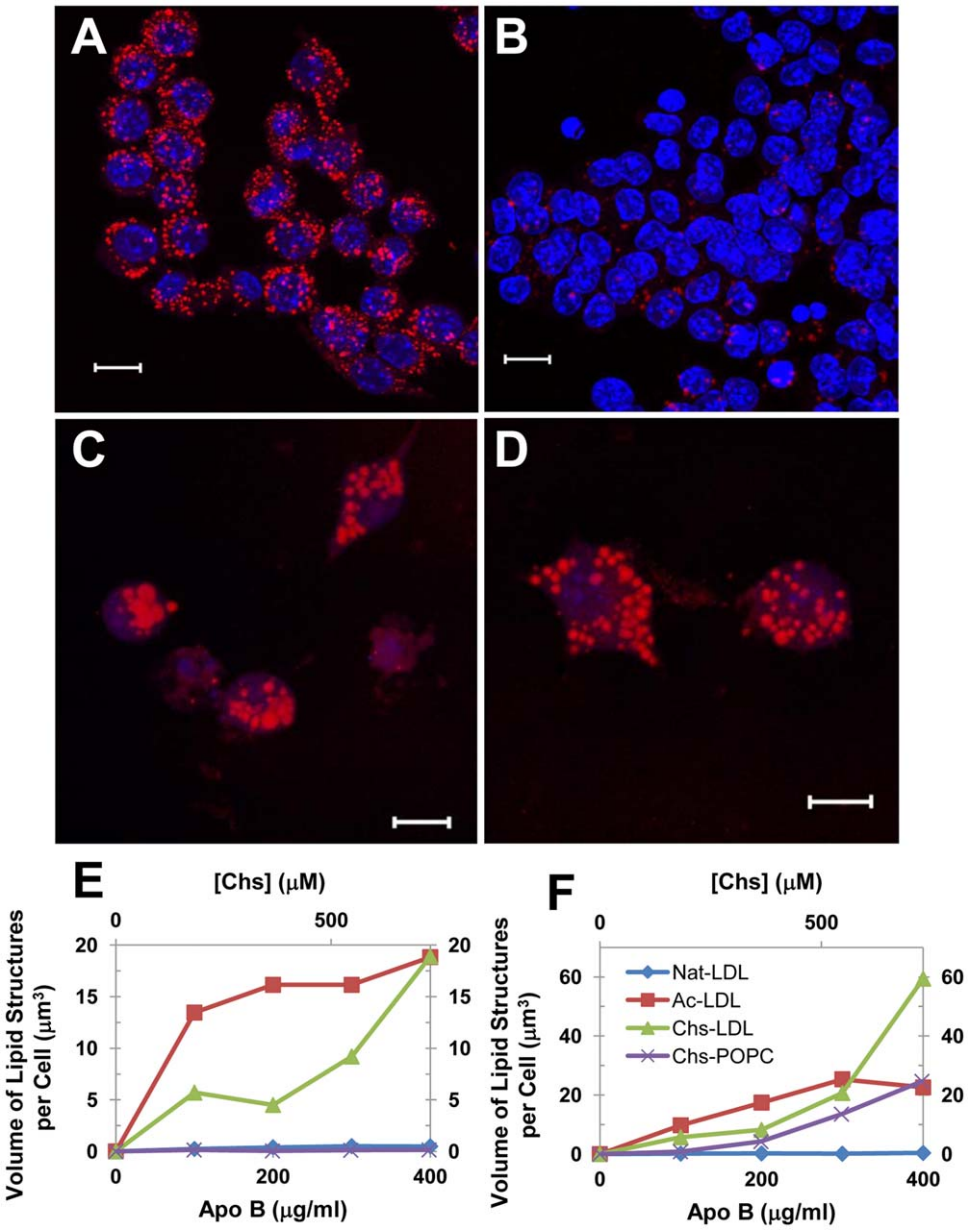
Addition of Chs-LDL to RAW cells induces the formation of large Oil Red-positive organelles. RAW cells were incubated for 48 h with Ac-LDL (A), Nat-LDL (B), Chs-LDL (1000∶1) (C), all at 400 µg LDL protein/ml; or Chs-POPC liposomes (45∶55) (D) at a particle density equivalent to that of Chs-LDL. After incubation the cells were fixed with PFA and stained with Oil-Red O and DAPI as described under Methods. Red stain, lipid organelles. Blue stain, nuclei. The images are projections of Z-stacks. Bars, 10 µm. Quantitative estimation of the total volume of lipid-rich structures per cell as a function of the Apo-B concentration (lower X-axis scale) or Chs (upper X-axis scale) concentrations after 24 h (E) and 48 h (F) incubations are also shown.

Macrophages and other sub-endothelial cells suffering from lipidosis are a defining characteristic of atherosclerotic plaques and are believed to be one of the first stages in atherogenesis. Ox-LDL are known to induce lipidosis in macrophages and subsequently cause apoptotic cell death due to uncontrolled uptake of lipid-rich particles combined with putative blockage of any one (or more) of several lipid processing reactions. Qualitatively, the process is morphologically similar to phenotypes seen in several lipid-storage pathologies [9]. In order to assess the ability of the Chs-LDL model to mimic this feature of Ox-LDL, RAW264.7 macrophages (referred to as RAW cells hereafter) were incubated with increasing amounts of Chs-LDL for 24 or 48 h and then fixed, stained with Oil-Red O or Bodipy, dyes that stain neutral lipid deposits in cells, and analyzed by confocal microscopy. Lipid loaded RAW cells resulted when these were exposed to Chs-LDL with a Chs/LDL ratio of 1000∶1 for 48 h (Figure 2C), the lower Chs∶LDL ratios did not produce any significant intracellular lipid accumulation. Therefore, only Chs-LDL (1000∶1) was used in further experiments. Since Ac-LDL are also known to rapidly produce lipid engorged cells [27], although the lipid is accumulated in storage organelles (lipid droplets) in this case and is, therefore, reversible [28], RAW cells were exposed to Ac-LDL as a positive control (Figure 2A), Nat-LDL as a negative control (Figure 2B), and Chs-POPC liposomes at a molar ratio 45∶55 (these liposomes have the same number of Chs molecules per particle as Chs-LDL (1000∶1)). After 48 h incubation with Chs-POPC liposomes RAW cells also exhibited lipid accumulation (Figure 2D) but less exuberantly than Chs-LDL. Since Nat-LDL in the absence of serum always induce a massive lipid accumulation in RAW cells, all experiments were carried out in the presence of serum.

The effect of Ac-LDL, Chs-LDL and Nat-LDL concentration on total volume of lipid deposits per cell as a function of time is shown in Figure 2E–F. Lipid accumulation was faster in macrophages incubated with Ac-LDL (lipid droplets were visible after 24 h incubation with any concentration, expressed as µg LDL apoprotein/ml, of Ac-LDL tested) than with any of the other LDL models and showed saturation behavior as expected for saturable receptor-mediated uptake [9] (Figure 2E, red curve). In contrast, exposure to Chs-LDL resulted in a continuous nonlinear non-saturating increase in neutral lipids inside the cell at concentrations between 100 and 400 µg/ml (see Figure 2E–F, green curves), the latter being the highest concentration tested. The results obtained with Chs-LDL at 400 µg/ml assume particular significance when it is considered that LDL concentrations in the arterial *intima* range from about 0.7 to 2.7 mg/ml [29], [30]. RAW cells incubated with Chs-POPC liposomes showed similar but much slower non-saturable lipid accumulation with increasing Chs concentration (expressed as µmol/liter of Chs in the incubation medium) (Figure 2F, purple curve) that became noticeable only upon incubation for 48 h, but reached a level that was only 42% of that observed upon incubation with Chs-LDL. Lipid accumulation in cells incubated with Nat-LDL was negligibly small (Figure 2E–F, blue curve). Clearly, the kinetics and, therefore, the mechanism of intracellular lipid accumulation into macrophages is LDL-model dependent.

Another striking difference between the lipid-rich structures in macrophages incubated with Ac-LDL and Chs-LDL was their average cross sectional areas – 0.8 µm^2^ and 3 µm^2^ in cells incubated with 400 µg/ml of Ac-LDL and Chs-LDL, respectively.

**Figure 3:**
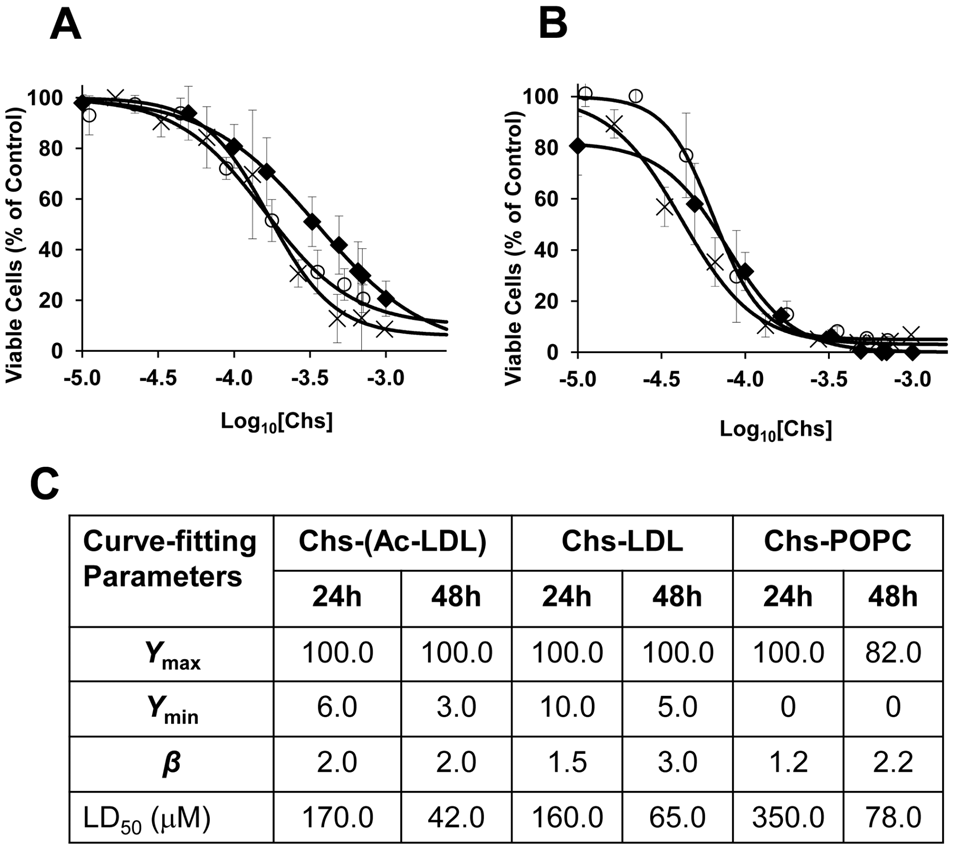
Chs affects the viability of RAW cells. Cells were exposed to different concentrations of Chs-LDL (1000∶1) (Ο), Chs-POPC liposomes (45∶55) (♦), and Chs-(Ac-LDL) (1000∶1) (X), for 24 (A) or 48 h (B) under the experimental conditions described in Methods. Cell viability was assessed by the MTT assay and is expressed relative to the viability of control cells exposed to Nat-LDL (for Chs-LDL) or Ac-LDL (for Chs-(Ac-LDL)). Control cells for Chs-POPC liposomes treated cells were incubated with POPC liposomes. Values are means ± SD of three separate experiments. Experimental data were fitted using a log-logistic equation: 
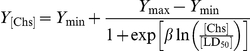
, where *Y*_[Chs]_, *Y*_max_ and *Y*_min_ are the fraction of viable cells for a given exposure concentration of Chs, the maximum, and the minimum values of *Y* required for the best fit, respectively, and *β* is the slope of the change between *Y*_max_ and *Y*_min_. The table (C) shows the fitting parameters (including LD_50_) obtained from the theoretical fits to the experimental data.

From confocal microscopy images, Figure 2, it was clear that Chs-LDL also induced massive apoptotic cell death (visualized by DAPI staining). Toxicity was measured by the MTT test, 24 (Figure 3A) and 48 h (Figure 3B) post-exposure to Chs-(Ac-LDL) (1000∶1 molar ratio), Chs-LDL (1000∶1 molar ratio), and Chs-POPC (45∶55 molar ratio) liposomes. The MTT test is based on the reduction of MTT to a formazan by intracellular metabolically derived reducing equivalents. Under our experimental conditions Ac-LDL, Nat-LDL, and POPC liposomes were not toxic towards RAW cells and were used as controls for Chs-(Ac-LDL), Chs-LDL and Chs-POPC liposomes, respectively. Cell viability is expressed as a percentage of the viability of control cells. The LD_50_, Chs concentration at which cell viability was 50% of the control, was determined for each exposure time (Figure 3C). Chs was toxic to RAW cells, the toxicity being dose- and time-dependent but only weakly vehicle-dependent (Figure 3A–C). These toxicity results are in agreement with the anti-proliferative and apoptotic activities of Chs in cancer cells [31].

It is of interest to note that while the theoretical curves fitted to the data in Figure 3C show that Chs-POPC treatment of cells (for 24 or 48 h) leaves no residual viable cells at “infinite" concentrations of Chs, both Chs-(Ac-LDL) and Chs-LDL treatment leaves a residual population of between 5 and 10% of cells alive (as judged by the MTT assay). This, for the present mysterious, observation may be related to macrophage sub-populations in which some sub-populations are more susceptible to acute Chs toxicity and others are not. We are presently investigating this possibility.

### Chs-LDL exposure leads to lysosomal lipid accumulation in RAW cells

**Figure 4:**
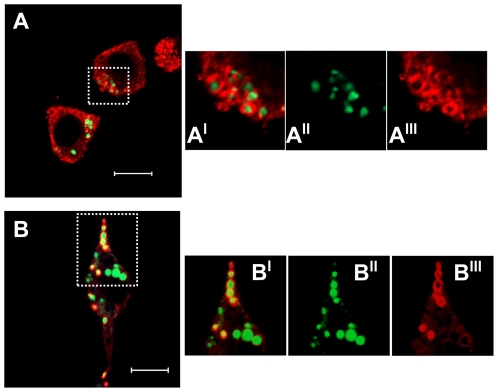
ADRP, a marker of lipid droplets, decorates the intracellular lipid structures induced by Chs-LDL. RAW cells were incubated for 48 h with Ac-LDL (A-**A^III^**) or Chs-LDL (B-**B^III^**). Panels (A) and (B) are confocal single-slice images and show the neutral lipids and the ADRP distribution. Neutral lipid-rich structures, in green, were stained with Bodipy 493/503. ADRP, in red, was visualized by immuno-staining with polyclonal antibodies as described under Methods. Panels (A) and (B) are merged images. In panels (A**^I^**) and (B**^I^**) the regions outlined with the rectangles in panels (A) and (B), respectively, are enlarged. Panels (A**^II^**) and (B**^II^**) show Bodipy staining 493/503, panels (A**^III^**) and (B**^III^**) show the distribution of ADRP. Bars, 10 µm.

**Figure 5:**
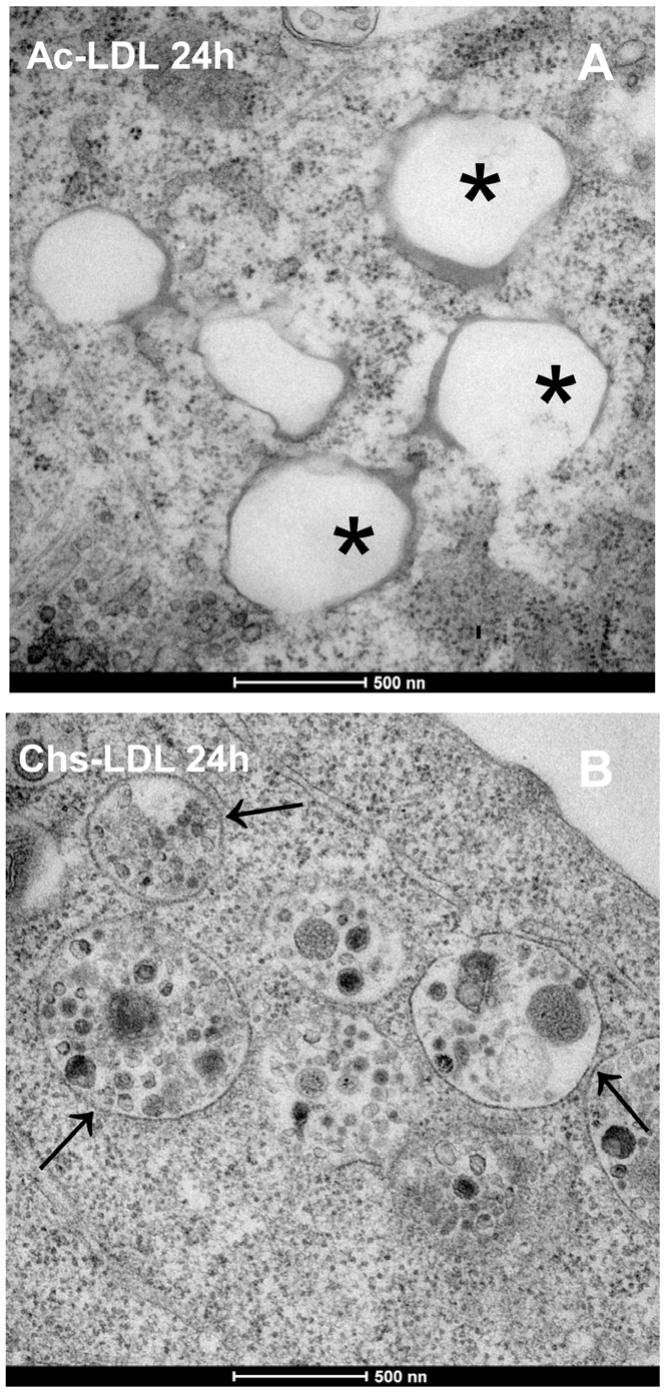
Chs-LDL induces the formation of bilayer vesicles full of electron dense material. Transmission electron microscopy of cells treated for 24 h with Ac-LDL (A) or Chs-LDL (B). Cytoplasmic lipid droplets (organelles with a single monolayer) and vesicular structures with a bilayer and with electron dense material are visible in cells treated with Ac-LDL and Chs-LDL, respectively. The asterisk (*) indicates cytoplasmic lipid droplets in (A). Arrows point to bilayer vesicles with electron dense material inside in (B). Bars, 500 nm.

**Figure 6:**
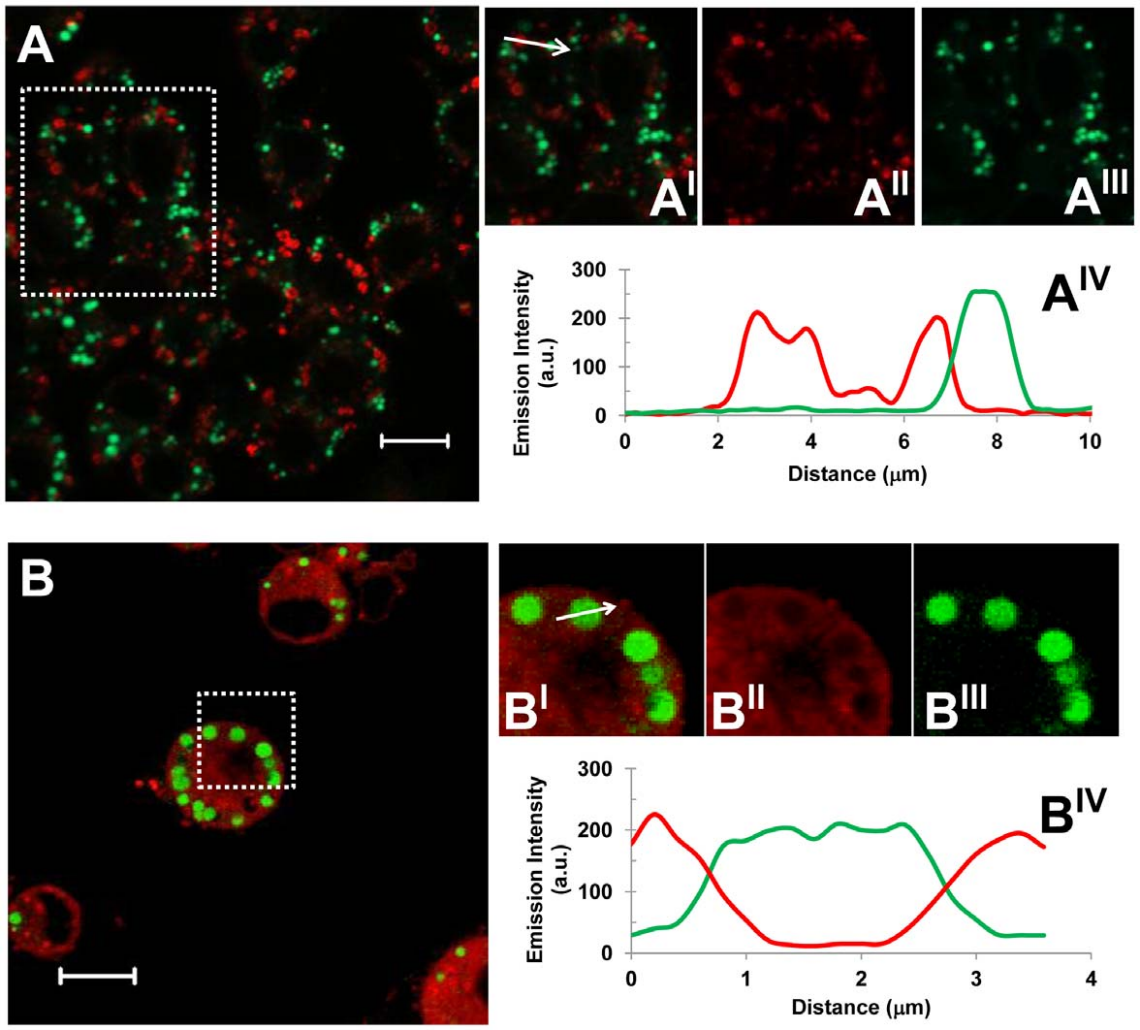
LAMP-2 decorates the neutral-lipid-rich vesicles in macrophages incubated with Chs-LDL. Macrophages incubated for 48 h with Ac-LDL (A-**A^IV^**) or Chs-LDL (B-**B^IV^**) were fixed and double labelled with anti-LAMP-2 antibodies and Bodipy. Merged images (panels (A-**A^I^**) and (B-**B^I^**)) show LAMP-2 in red and Bodipy in green. Panels (A**^I^**) and (B**^I^**) are the zoomed regions outlined by the rectangles in panels (A) and (B), respectively. Panels (A**^II^**) and (B**^II^**) show LAMP-2 staining. Panels (A**^III^**) and (B**^III^**) show lipid droplet staining. All panels show confocal single slice images. Asterisks point to LAMP-2-positive lipid droplets. Bars, 10 µm. The graphs (A**^IV^**) and (B**^IV^**) show relative intensity scans along the arrows in panels (A**^I^**) and (B**^I^**), respectively, of the LAMP-2 (red line) and Bodipy (green line) stains.

Most mammalian cells package neutral lipids into storage droplets that are surrounded by a monolayer of phospholipids and a specific set of proteins including the adipose differentiation-related protein (ADRP or adipophilin) [32]. The lipid structures formed in RAW cells were characterized by incubating the macrophages with the different LDL models for 48 h, followed by fixing and staining with Bodipy for neutral lipids and with an antibody for ADRP. At first sight, the lipid-rich structures in macrophages incubated with Ac-LDL and Chs-LDL were both decorated with ADRP (Figure 4A-A^III^ and B-B^III^, respectively). However, in cells incubated with the Ac-LDL, ADRP was partially localized in the cytosol (Figure 4A-A^III^), whereas in cells incubated with Chs-LDL (Figure 4B-B^III^) ADRP was almost totally localized around the neutral lipid structures. More detailed differences between the lipid structures were identified by transmission electron microscopy. At the ultrastructural level RAW cells incubated with Chs-LDL were full of large vesicles with electron dense material inside and surrounded by a lipid bilayer (Figure 5B, arrows) while the cells incubated with Ac-LDL showed cytosolic lipid-storage droplets (Figure 5A). Co-localization studies in Chs-LDL treated RAW cells for 48 h showed that the large Bodipy-positive vacuoles were decorated on the surface by the Lysosomal Associated Membrane Protein-2 (LAMP-2), a late endosome/lysosome marker (see Figure 6B-B^III^ and the graph with the fluorescence distribution, Figure 6B^IV^). This feature was never observed in macrophages incubated with Ac-LDL (Figure 6A-A^III^ and A^IV^).

Thus, RAW cells exposed to Chs-LDL accumulate neutral lipids in late endosome/lysosome structures (similar to Ox-LDL treated macrophages [9]) and not in cytosolic lipid storage droplets as in the case of Ac-LDL treated cells [28]. The phenotype we observe resembles the pathology seen in the Niemann-Pick C syndrome (a lysosomal lipid-storage disease), also characterized by an aberrant lysosomal accumulation of lipids.

Chs-LDL mimics two important features of the Ox-LDL effect on macrophages: lipid accumulation in a late endosome/lysosome compartment and apoptotic cell death.

### Cholesteryl esters accumulated in RAW cells are different when exposed to Chs-LDL and to Ac-LDL

Total lipid extracts of macrophages engorged with neutral lipids after Chs-LDL and Ac-LDL treatment were initially analyzed by thin layer chromatography (Methods S1, Figure S1). As expected from reports by others [28], Ac-LDL treatment of RAW cells led to accumulation of cholesteryl esters. Chs-LDL treated macrophages also accumulated large amounts of cholesteryl esters but a small difference in TLC migration patterns of the cholesteryl esters of the two groups suggested that they were chemically different.

These differences, apparent in TLC analysis, were quantitatively confirmed by high resolution shotgun lipidomics MS analysis [33] of the total lipid extracts of cells incubated with Ac-LDL or Chs-LDL for 24 and 48 h incubation (Table 1). 17 different cholesteryl esters were identified and quantified of which the most abundant are listed in Table 2. The cholesteryl esters of lipid extracts from macrophages incubated with Ac-LDL and Chs-LDL for 24 and 48 h were very significantly different. In both groups of treated cells the most abundant cholesteryl esters were cholesteryl oleate (18∶1) and cholesteryl linoleate (18∶2) but cholesteryl linoleate was the predominant ester (50.6±1.1% of the total cholesteryl esters) in Chs-LDL treated cells whereas cholesteryl oleate predominated (40.2±0.7%) in Ac-LDL treated cells. Since cholesteryl linoleate is the predominant cholesteryl ester (57.1±2.5%) in Nat-LDL (see Table 2), it appears that the cholesteryl esters accumulated in Chs-LDL treated RAW cells were simply an unprocessed accumulation of the cholesteryl esters of the endocytosed LDL particles. In Ac-LDL treated RAW cells, however, ingested cholesteryl esters were processed before being stored in lipid droplets, as expected for normal cholesterol homeostasis in macrophages [1].

**Table 1:** Lipid composition of RAW cells subjected to various treatments.

	Polar Lipids								Apolar Lipids			
Treatment of Cells:	Total Polar Lipid		Free Cholesterol		Chs		Free Chol+Chs		Cholesteryl Esters		Triacylglycerides	
	24 h	48 h	24 h	48 h	24 h	48 h	24 h	48 h	24 h	48 h	24 h	48 h
Control	100±10	100±8	22±3	25±1	0	0	22±3	25±1	16±4	14±3	1.6±0.3	2.7±1.8
Ac-LDL	100±13	100±15	26±2	30±2	0	0	26±2	30±2	61±7	58±8	2.9±0.4	6.7±2.8
Chs-LDL	100±21	100±13	25±5	35±3	29±1	43±1	54±6	78±4	24±16	58±16	3.0±0.7	4.3±2.4
Chs-POPC	100±18	100±20	14±1	14±3	40±1	57±1	54±2	71±4	5±2	5±2	2.1±0.3	1.8±0.9
Native LDL	100±4		43±1		0		43±1		257±25		10.3±1.0	

Polar lipids have been defined as the sum of phosphatidylcholines, phosphatidylethanolamines, sphingomyelins, ceramides, diacylglycerides, free cholesterol and cholesteryl hemisuccinate. All values are expressed as a mol % of the total polar lipids. The apolar lipids (cholesteryl esters and triacylglycerides) are expressed as a mol % of the total polar lipid fraction. For purposes of comparison, the bottom row shows the lipid composition of Nat-LDL. Chs-LDL are Nat-LDL loaded with Chs with a Chs/LDL particle molar ratio of 1000∶1, Chs-POPC are POPC liposomes with a Chs/POPC molar ratio of 45∶55. The values in the columns entitled “Total Polar Lipids" is a reference point by definition and, therefore, always 100%. These values have only been included to show the standard deviation of the measurement since all the other values are given relative to the “Total Polar Lipid" content.

**Table 2:** Fatty acid composition of the cholesteryl esters extracted from RAW Cells and, for purposes of comparison, the fatty acid composition of Nat-LDL.

Fatty Acid	Nat-LDL		Ac-LDL		Chs-LDL		Extract of Nat-LDL
	24 h	48 h	24 h	48 h	24 h	48 h	
14∶0	4.3±0.6	2.4±0.4	9.8±0.7	8.5±1.6	4.7±1.7	1.5±0.1	0.6±0.2
16∶0	6.3±1.8	5.0±1.7	18.2±1.3	14.2±2.2	14.1±1.7	12.9±1.0	9.1±1.2
16∶1	1.7±0.1	1.6±0.5	4.3±0.2	4.3±0.6	4.7±0.7	4.1±0.4	2.8±0.4
18∶0	2.8±1.1	1.4±0.2	9.2±0.4	7.0±0.9	3.0±1.6	1.0±0.2	0.4±0.1
18∶1	**13.7±1.7**	**13.3±2.0**	**39.2±0.9**	**40.2±0.7**	**27.3±4.8**	**21.3±1.6**	**15.3±1.4**
18∶2	**4.5±0.3**	**8.7±5.9**	**11.1±1.3**	**15.8±0.7**	**37.8±9.2**	**50.6±1.1**	**57.1±2.5**
20∶4	0.7±0.1	2.6±1.3	1.4±0.6	2.4±1.6	3.9±1.1	5.2±1.3	9.5±0.7

Quantitative lipidomic analysis of the intracellular cholesteryl esters in lipid extracts of cells incubated for 24 or 48 h with 300 µg/ml of Ac- or Chs-LDL. Only the major cholesteryl esters are represented in the table. Changes in the content of Cholesteryl oleate (18∶1) and Cholesteryl Linoleate (18∶2) are highlighted. Lipid extracts of native LDL were used as control. The cholesteryl esters distribution is shown as percentage of total cholesteryl esters. The results are the mean ± SD of three independent experiments obtained with three different LDL preparations.

Thus, post-internalization processing of LDL particles when macrophages are exposed to Chs-LDL is blocked at the late endosome/lysosome stage and the cholesteryl esters accumulate in these organelles, indicating a pathology in cholesterol homeostasis.

### Chs is internalized mainly in a vehicle-independent manner, possibly via passive diffusion

**Figure 7:**
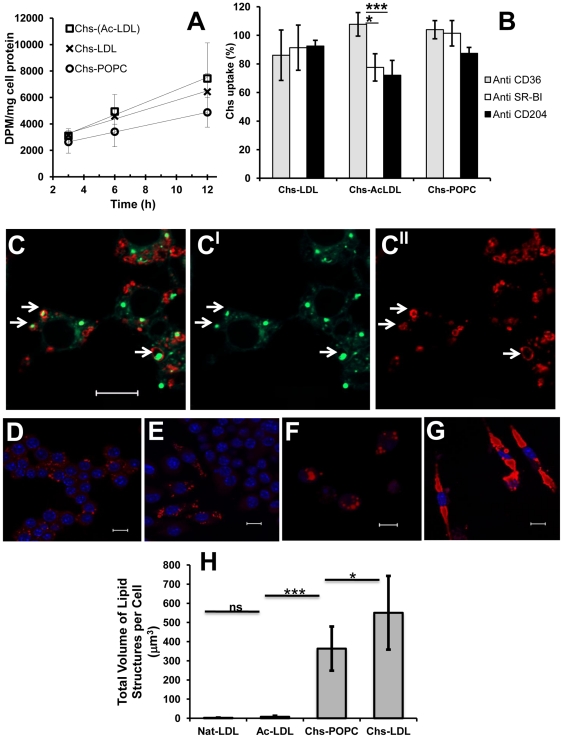
Chs is internalized mainly by passive diffusion. Panel (A) shows the incorporation of intracellular radioactivity as a function of time when RAW cells were exposed to Chs-LDL (×); Chs-(Ac-LDL) (□); and Chs-POPC liposomes (○), with similar ^3^H-Chs levels. After incubation the cells were acid washed and then scraped, and finally the radioactivity and protein were quantified as described under Methods. Panel (B) shows the effect of specifically blocking the scavenger receptors CD36, SRBI and CD204 by first exposing the RAW cells to specific inhibitory antibodies for 15 min on ice followed by raising the temperature to 37°C and exposing the cells to ^3^H-Chs-LDL, ^3^H-Chs-(Ac-LDL), both at 300 ug/ml Apo-B, or ^3^H-Chs-POPC liposomes. The Chs concentration and the ^3^H radioactivity was similar in all Chs-containing particles. Results are expressed as percentages relatively to the control cells (treated similarly but with non-inhibitory antibodies). After incubation with the Chs-containing particles the cells were processed as described above. Chs uptake results were expressed as percentages relative to the control. The results are the mean ± SD of three independent experiments. **, p<0.01; *, p<0.05. Panel (C-**C^II^**) shows that RAW cells pre-incubated with Chs-POPC liposomes during 16 h and subsequently exposed to Nat-LDL accumulate neutral lipids in the lysosomal compartment. RAW cells were incubated with Chs-POPC liposomes during 16 h and then with Nat-LDL (300 µg/ml) for 48 h in absence of liposomes. The cells were then fixed and double labeled with Bodipy (C**^I^**) for neutral lipid staining and anti-LAMP-2 antibody (C**^II^**). The merged image (C) shows Bodipy staining in green and LAMP-2 staining in red. Arrows point to LAMP-2-positive cytosolic structures that contain neutral lipids. Bar, 10 µm. Panels (D–**G**) show that the lysosomal accumulation of neutral lipids induced by pre-treatment of RAW cells with Chs-LDL is irreversible. Raw cells were pulsed with 300 µg/ml of Ac-LDL (panel **D**) or Chs-LDL (panel **F**) for 48 h and then chased for 96 h. Cells pulsed with Ac-LDL (D) and then chased (E). Cells pulsed with Chs-LDL (F) and then chased (G). Lipid-rich structures visualized by Oil-red staining (red), DAPI staining (blue). All are merged images. Bars, 10 µm. The total volume of lipid structures per cell, quantified after the 96 h chase, is shown in panel (H). The results are the mean ± SD of three independent experiments. In every experiment 20 individual cells were analyzed. ***, p<0.0001; *, p<0.05; ns, not significant.

To elucidate the mechanisms of Chs entry, macrophages were treated for 3 to 12 h with radioactively labeled Chs in Chs-(Ac-LDL), Chs-LDL, and Chs-POPC liposomes with similar total Chs concentrations. The intracellular radioactivity was examined as a function of incubation time (Figure 7A). Surprisingly, Chs incorporation into the cells was only weakly vector dependent (only slightly faster for Chs-(Ac-LDL) and Chs-LDL treated cells as compared with Chs-POPC liposome-treated cells) suggesting that Chs entered the cells mainly *via* passive diffusion and trans-membrane translocation between the Chs-containing particles and the cells. One scenario for this process could be a rapid equilibrium of the type:
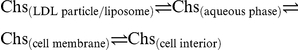
This equilibrium only becomes possible because Chs is amphiphilic with an appreciable, albeit low, solubility in the aqueous phase.

To further clarify this apparent vector-independent Chs uptake, we examined the effect of blocking scavenger receptors in RAW cells that are known to play a role in the uptake of modified LDL, namely, CD36, SRA (CD204) and SR-BI [34]. We compared Chs uptake by RAW cells in the presence and absence of receptor-blocking antibodies as described in [35]. Chs uptake was not affected by any of the scavenger receptor-inhibiting antibodies when macrophages were exposed to Chs-POPC liposomes or to Chs-LDL for 3 h (Figure 7B). However, a 22.4% (p<0.05) and 27.9% (p<0.1) reduction of Chs uptake was observed when macrophages were exposed to Chs-(Ac-LDL) in the presence of anti-CD204 or SR-BI inhibitory antibodies. This result suggests that in addition to passive diffusion, endocytosis of Ac-LDL *via* CD204 and SR-BI receptors, but not CD36, contributes in a small measure to Chs internalization *via* the Ac-LDL vehicle [1]. A similar mechanism involving some (as yet) unidentified receptor may also be involved in Chs-LDL treated cells.

Because, Chs gets into the cells mainly by passive diffusion and Chs-LDL appear to be poor substrates for lysosomal enzymes (Table 2) we hypothesized that Chs accumulates in the endolysosomal membranes, changing their biophysical/biochemical properties, and entering cargo (independent of the endocytic receptor) is retained within this organelle because it cannot be degraded. This hypothesis was confirmed by incubating RAW cells with Chs-POPC for 16 h and then exposing the cells to Nat-LDL in the absence of Chs for 48 h. The macrophages exhibited intracellular lipid accumulation in large structures surrounded by LAMP-2 resembling the phenotype obtained after Chs-LDL treatment (Figure 7C-C^III^), a phenotype that was not seen when RAW cells were exposed to Nat-LDL without any prior exposure to Chs.

### Endolysosomal lipid accumulation induced by Chs-LDL is irreversible

The last question we addressed was how macrophages handled the accumulated lipid in the various cases examined in this work. Reversibility of lipid accumulation in RAW cells was assessed by pulse-chase experiments (Figure 7D–H). In cells exposed for 48 h to Ac-LDL a 96 h chase did not fully deplete the cells of lipid droplets, but it was obvious that these cells had much less neutral lipids than before the chase (Figure 7D–E). In contrast, cells incubated for 48 h with Chs-LDL and then chased for 96 h in the absence of these particles exhibited a striking phenotype. The cells seemed completely full of giant lipid-rich structures (Figure 7F–G).

These results suggest that lipid accumulation in endolysosomes within the cells exposed to Chs (Figure 7H) is an irreversible “one way" process – lipid accumulation is continuous and the accumulated lipid is neither available for utilization by the cell nor for efflux. Interestingly, and once again, this feature is also observed with Ox-LDL ([36], [37] and for review see [9]).

## Discussion

The induction of lipidosis in macrophages in the *intima* is generally accepted to be one of the initial steps of atherogenesis in the arterial wall. These cells that originate atheromata have a defective cholesterol homeostasis that results in irreversible accumulation of lipid in endolysosomes, a property that is characteristic of many endolysosomal lipid storage diseases. Why exactly this happens is debated but there seems to be a consensus that the process involves defective post-internalization handling by macrophages (and other cells) of trapped and physico-chemically modified LDL in the subendothelial space.

One of the chemical modifications of LDL that has received much attention is the oxidative modification of their linoleic and arachidonic acid-containing components. Oxidative breakdown of either of these fatty acids produces a very large number of products many of which are aldehydes. In this category of products 4-hydroxynon-2-enal (HNE) and malondialdehyde (including its protein conjugates) have been most extensively studied. But it is also known that HNE is very rapidly (upto >90% in 3 min, depending on cell type) converted by cells to other products, the principal one being 4-hydroxynon-2-enoic acid [38]. Cholesteryl linoleate is the most abundant cholesteryl ester in LDL. Oxidative scission of this cholesteryl ester that produces HNE produces an equimolar amount of cholesteryl-9-oxononanoate which, like HNE, should rapidly be further oxidized in an oxidizing environment or through the action of intracellular aldehyde oxidases to cholesteryl azelate, a cholesteryl hemiester of a short (C_9_) chain dicarboxylic acid. Several ω-oxoester precursors of hemiesters (of C_4_ through C_9_ diacids) of cholesterol have been identified in Ox-LDL [26]. Cholesteryl-4-oxo-butanoate, the precursor of Chs, is one of them. Cholesteryl hemiesters have been identified as ligands of the plasma protein, β_2_-glycoprotein-1, and shown to be involved in the formation of atherogenic complexes of this protein with Ox-LDL [22], [23]. These complexes in their turn have a positive correlation with disease severity as well as increased risk for adverse outcomes in patients with acute coronary syndromes [24], [25]. It may, therefore, be assumed that cholesteryl hemiesters are relevant *in vivo* for atherogenesis.

Ox-LDL are known to induce lipidosis with irreversible endolysosomal accumulation of lipid from macrophages in vitro [9], [36], [37], [39]. However, due to the compositional complexity of Ox-LDL it becomes impossible to establish etiological hierarchies among the chemical constituents of Ox-LDL with regard to the induction of lipidosis.

In consideration of the above, we decided to develop a model Nat-LDL particle enriched in a single chemical constituent of Ox-LDL that could possibly be responsible for the induction of lipidosis. We demonstrate this principle here with native non-oxidized LDL enriched in Chs, a cholesteryl hemiester (of a C_4_ diacid) model for the several hemiesters of cholesterol that can be formed in Ox-LDL. These hemiesters are all amphiphilic, have a negative charge at physiological pH and can be expected to partition favorably into cell membranes; they equilibrate through passive diffusion between the Ox-LDL (where they are formed) and the aqueous (extra- and intracellular) phases, as well as all membranes of neighboring cells. Cholesteryl hemiesters, or for that matter, their precursor ω-oxoacid esters of cholesterol, have been mostly ignored as atherogenic agents in the literature although the other scission product of cholesteryl linoleate, HNE, has received abundant attention. The methodology we describe here may be profitably used to study the ability of several other components of Ox-LDL (isolated or in controlled combinations) to induce lipidosis with irreversible endolysosomal lipid accumulation in macrophages and other sub-endothelial cells. The model may, in principle, be useful in studying detailed kinetics of the phenomenon as well as to study synergistic and/or antagonistic processes among the chemical constituents of oxidized, or otherwise modified, LDL. We have previously described strategies for enriching lipid aggregates (including LDL) with different kinds of amphiphiles [40], [41].

The effects of Chs on RAW cells may be summarized as follows: 1) Formation of lipid loaded cells with lipid accumulation in the endolysosomal compartment; 2) Incapacity of affected cells to metabolize (hydrolyze and re-esterify) internalized cholesteryl esters; 3) Irreversible and uncontrolled lipid accumulation in the endolysosomal compartment; and 4) Apoptotic cell death.

We have no data that informs us as to exactly why Chs has these effects. The reasons may be complex but we shall speculate on this theme. We have shown that the internalization of Chs is largely a vehicle-independent process, probably occurring mostly *via* passive diffusion and equilibration between the LDL particle and its immediate environment (including the aqueous phase and all neighboring cells). Chs is an amphiphile which, due to its cholesterol moiety, partitions very favorably into membranes. The simplest effect that can be imagined is an increase in membrane order, similar to the effect of cholesterol [18], [42]. Changes in membrane order are known to affect enzyme activity including the activity of the lysosomal H^+^-ATPase which is responsible for acidification of lysosomes [43]. In principle, at least two membrane loci can be imagined where incorporation of Chs would cause perturbations of membrane physiology – the endosome/lysosome membrane and the membrane of the endoplasmic reticulum. In the former, changes in the pH of the endolysosomal compartment and consequent modulation of hydrolytic enzyme activities may be expected. The endoplasmic reticulum is the locus of Acyl-CoA-cholesterol acyltransferase (ACAT) activity and inhibition of this enzyme (assuming that cholesteryl esters continue to be hydrolyzed in the endolysosomes) would result in a continuously increasing intracellular free cholesterol concentration with a consequent stiffening of all cellular membranes including that of lysosomes so that, eventually, this compartment would also malfunction. Our results (Table 2) indicate that the internalized cholesteryl linoleate in cells exposed to Chs-LDL is not hydrolyzed in the endolysosomal compartment where it accumulates, whereas those exposed to Ac-LDL hydrolyze the internalized cholesteryl esters, re-esterify the cholesterol to cholesteryl oleate and store it in lipid droplets that can be depleted in the normal processes of cholesterol homeostasis. Also, the total (free cholesterol+Chs) content of Chs-treated cells is more than twice the free cholesterol content of control cells or cells treated with Ac-LDL (Table 1).

Clearly the effects observed by us are not simply due to ingestion of a large amount of cholesteryl esters by the cell – the cholesteryl esters of Ac-LDL, which are internalized more rapidly than Chs-LDL, are processed normally and do not create a pathological state. The pathological state is caused by the Chs in Chs-LDL. Our results (Table S1) indicate that Chs is very slowly (if at all) hydrolyzed in the RAW cells. It, therefore, accumulates with time and the cells' incapacity to handle the accumulated Chs is probably responsible for the cell damage observed here. We are further investigating the details of this process. Artificially increased levels of cellular free cholesterol are known to cause lipidosis in macrophages [44], [45]. We propose that cholesteryl hemiester accumulation within cells in the vicinity of Ox-LDL (or by cells that internalize Ox-LDL) in the arterial intima may be at least one of the processes that is responsible for atherogenesis.

We recognize that the Chs concentrations to which the RAW cells were subjected in this work may be much higher than can be accounted for under physiological conditions. However, the spontaneous oxidation of Nat-LDL in cell culture media imposes limits on exposure times and, therefore, requires higher doses. A more detailed study of the dose and exposure-time dependence of the effects we report here may be warranted in the future.

Finally, we note with particular interest, that whereas the large majority of the RAW cells subjected to Chs exposure died apoptotically, a small subset, representing between 5 and 10% of the population, survived and seemed to accumulate lipid progressively and irreversibly in the endolysosomal compartment. This observation may be related to macrophage heterogeneity [43]. If so, it will be of interest to know exactly which subset of a macrophage population forms atherogenic cells. This could be one of the keys to eventual prophylactic therapies.

## Materials and Methods

### Chemicals and antibodies

Oil-red, Cholesteryl hemisuccinate, Dextran (mol wt 9,000–11,000) were obtained from Sigma. PO-PC and cholesterol were purchased from Avanti Polar Lipids (Alabaster, AL). [^3^H]-Colesterol was from GE Healthcare. The other chemicals used were of analytical grade from local sources.

Rhodamin-phalloidin, Bodipy 493/503 and FITC-Dextran were purchased from Molecular Probes. DAPI was from Fluka. The anti-mouse (ABL-93) Lamp-2 antibody was from the Developmental Studies Hybridoma Bank, (University of Iowa, Iowa City, IA). Polyclonal guinea-pig anti-ADRP antibody was from Progen Biotechnik (Heidelberg, DE). Secondary antibodies were from Molecular Probes or from Jackson Immunoresearch. Mouse Anti-mouse CD36 (552544) and the negative control mouse anti-mouse IgA (553476) were purchased from BD Pharmigen; rat anti-mouse CD204 (MCA1322) and the negative control rat anti-mouse g2b (MCA1125) were purchased from AbD Serotec. Rabbit anti-mouse SR-BI (NB400-113) and the negative control rabbit anti-mouse IgG (NB810-56910) were purchased from Novus Biologicals.

### Preparation of liposomes

Aqueous suspensions of lipids were prepared by mixing POPC and Chs at the desired ratios in an azeotropic mixture of chloroform and methanol. The solvent was evaporated by blowing dry nitrogen over the heated (blowing hot air over the external surface of the tube) solution and then leaving the residue in a vacuum desiccator for at least 12 h at 23°C. The dry residue and the hydration solution (20 mM Hepes, 0.11 M NaCl, 1 mM EDTA, pH 7.4) with or without Dextran in an amount necessary to obtain a density of 1.044 g/ml, were preheated in a water bath at 65°C. The samples were submitted to several cycles of vortex/incubation/mild sonication at 65°C for at least 1 h. The resulting multilamellar vesicle (MLV) suspensions were extruded through two stacked polycarbonate filters (Nucleopore) with a pore diameter of 0.1 µm using a minimum of 6 passes. During the extrusion the water-jacketed extruder (Lipex Biomembranes, Vancouver, British Columbia, Canada) was maintained at 65°C. Final lipid molar ratios of Chs/POPC were: 0/100, 70/30, and 45/55.

### LDL isolation, acetylation and Chs incorporation

Human LDL were isolated using a Beckman L80 ultracentrifuge equipped with a 70.1 Ti fixed angle rotor as described previously [46]. LDL were acetylated by repeated additions of acetic anhydride as described [47]. Chs-LDL were prepared by incubating Nat-LDL with Chs-POPC (70∶30) liposomes in Hepes buffer containing dextran (density of 1.044 g/ml) overnight at different LDL/Chs ratios at RT without stirring. After incubation the Chs-LDL were re-centrifuged to eliminate the remaining Chs-POPC liposomes and after dialysis they were passed through a 0.2 µm filter. Chs content of Chs-LDL was assessed by measuring the radioactivity of the samples. All stock and working solutions of different LDL models were stored under N_2_ at 4°C for a maximum of 2 weeks.

### Agarose gel electrophoresis

Electrophoresis of LDL preparations was carried out in 0.5% agarose gels in barbital buffer, pH 8.6, at a constant voltage of 75 V for 45 min, and LDL were stained with 0.5% Paragon Blue in 5% acetic acid for 3 min at room temperature. The gel was destained at room temperature with 5% acetic acid, followed by 20% acetic acid, 30% methanol. In each agarose strip, Nat-LDL were used as a control.

### Cell culture and incubation with the different LDL models and Chs-POPC liposomes

RAW 264.7 cells (ATCC) were maintained in DMEM supplemented with 10% fetal calf serum, 100 U/ml of penicillin and streptomycin. Cells were grown in a humidified incubator at 37°C under 5% CO_2_ and used for assays during the exponential growth phase. For lipid droplet staining RAW cells were grown on glass-coverslips or in 24 well-plates for 24 h. They were then incubated with Nat-LDL; Ac-LDL, Chs-LDL, Chs-(Ac-LDL) or Chs-POPC liposomes for different periods of time and at different concentrations, as indicated in the figure legends. After incubation the cells were fixed with PFA.

### Immunofluorescence and Confocal microscopy

Cells were grown on 24-well plates and fixed with 4% PFA for 60 min followed by quenching of the aldehyde groups with glycine or ammonium chloride and permeabilization with isopropanol (60%) or saponin (0.1%) for LAMP-2 and ADRP staining, respectively. Cells were then incubated with the primary antibodies for 1 h at room temperature, washed, and finally incubated with the secondary antibodies conjugated with a fluorophore for another 1 h. Antibody dilutions were 1∶100 for primary antibodies and 1∶500 for secondary antibodies conjugated with Cy3 or 1∶200 for secondary antibodies conjugated with Cy5 or Alexa Fluor®594. Fixed samples were analyzed by using the LSM 510 META point-scan confocal laser microscope (Zeiss) with a 63× oil-immersion objective.

### Neutral lipid staining and quantification

Neutral lipids were stained either with Oil-Red O (in 60% isopropanol, prepared from a stock solution at 0.4% w/v in 100% isopropanol) for 10 min or with Bodipy 493/503 (diluted 1∶1000 in PBS from a saturated ethanolic solution of Bodipy) for 15 min. DAPI at a final concentration of 30 nM was added for 20 min at room temperature to visualize nuclei. Quantification of the number and size of the lipid-rich structures was performed using ImageJ software.

### Electron Microscopy

Cells were seeded on glass coverslips and treated the next day. Cells were fixed in 2.5% glutaraldehyde in cacodylate buffer and processed for standard Epon embedding. 70 nm sections were analyzed in a TECNAI12 Biotwin electron microscope equipped with a bottom-mount 4×4 k CCD camera (FEI company).

### Toxicity evaluation by the MTT test

Cell viability was measured by the MTT assay as previously described [48].

### Mass Spectrometry

Cells were incubated in 6-well plates with Nat-LDL, Ac-LDL, Chs-LDL or Chs-POPC liposomes for 24 and 48 h. After incubations cells were washed three times with 150 mM ammonium bicarbonate and harvested from wells by scraping into 1 ml of ammonium bicarbonate. Protein content of the suspensions was evaluated and aliquots of the cell lysates or Nat-LDL suspensions containing 10 and 1 µg of protein, respectively, were extracted as described previously [49]. For absolute quantification, internal standards were added to the samples prior to lipid extraction. Cholesterol was quantified as described by Sandhoff *et al.* [50]. Top-down shotgun analysis was performed on a LTQ Orbitrap XL mass spectrometer (Thermo Fisher Scientific, Bremen, Germany) equipped with a TriVersa NanoMate robotic nanoflow ion source (Advion BioSciences, Ithaca, NJ) as described in [33]. Lipids were identified and quantified by LipidXplorer software developed in house [51].

### Chs uptake

After incubating the macrophages with ^3^H-Chs-LDL, ^3^H-Chs-(Ac-LDL), and ^3^H-Chs-POPC liposomes with similar radioactivity for different periods of time at 37°C, cell-associated [^3^H]-Chs was determined after rinsing macrophages with DMEM with 0.5% BSA at pH 3.4 for 10 min on ice, three further rinses with PBS, and a final rinse in distilled water. Macrophages were scraped off into 1 ml distilled water. Then cell samples were assayed for ^3^H radioactivity and for cellular protein.

For the experiments with the inhibitory antibodies the cells were incubated at 4°C for 15 min with 10 µg/ml of anti-CD36, anti-SR-BI, anti-CD204 and their respective antibody controls; after which 300 µg/ml of ^3^H-Chs-(Ac-LDL), ^3^H-Chs-LDL, or ^3^H-Chs-POPC liposomes (45∶55) with equivalent amounts of Chs were added to cells. The cells were then shifted to 37°C for 3 h (in presence of the inhibitory antibodies). After incubation the cells were treated as described above and the radioactivity and the cell protein quantified.

### Protein assay

The protein content of cell extracts was measured by the bicinchoninic acid (BCA, Pierce) assay using bovine serum albumin as a standard.

### Statistical analysis

Results are expressed as the means ± standard deviations (S.D.). Statistical significance was assessed by the Student *t-test* (two-tailed). A *p* value of <0.05 was considered to be statistically significant.

## Supporting Information

Figure S1 **Chs-LDL are poorly degraded within the lysosomal structures.** (a) Macrophages were incubated for 24 h with 300 µg/ml of Nat-LDL (lane 1), Ac-LDL (lane 2) or Chs-LDL (lane 3). In lane 4 lipid extracts of cells incubated with Chs∶POPC liposomes (45∶55) were loaded. At the end of the incubation time the lipids were extracted and resolved by TLC as in Methods S1. In each lane 60 µg of cell protein was loaded.
(PDF)

Methods S1 **Synthesis of ^3^H-Chs. Lipid extraction and preparative TLC.** Intracellular Chs hydrolysis.
(PDF)

Table S1 **Hydrolysis and re-esterification of cholesterol derived from ^3^H-Chs in RAW cells.** RAW cells were treated for 24 and 48 h with ^3^H-Chs-POPC liposomes (45∶55). The compounds were separated by TLC prior to measurement of radioactivity.
(PDF)
