# Supplementary figures and images for: Molecular Etiology of Atherogenesis – In Vitro Induction of Lipidosis in Macrophages with a New LDL Model
Source: PLoS One. 2012 Apr 13;7(4):e34822. doi: 10.1371/journal.pone.0034822 (PMC3325953; doi:10.1371/journal.pone.0034822)

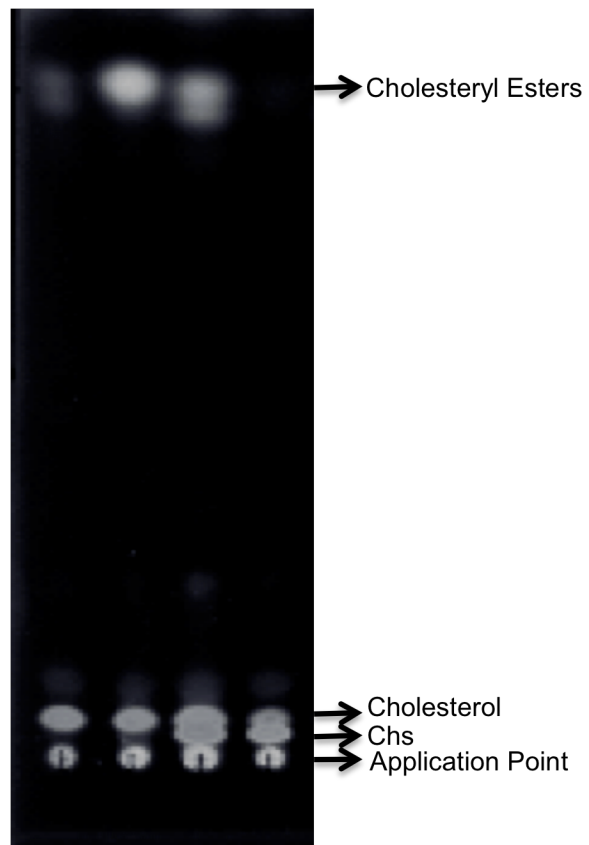

Supplement: Figure S1 — Chs-LDL are poorly degraded within the lysosomal structures. (a) Macrophages were incubated for 24 h with 300 µg/ml of Nat-LDL (lane 1), Ac-LDL (lane 2) or Chs-LDL (lane 3). In lane 4 lipid extracts of cells incubated with Chs∶POPC liposomes (45∶55) were loaded. At the end of the incubation time the lipids were extracted and resolved by TLC as in Methods S1. In each lane 60 µg of cell protein was loaded. (PDF) [file pone.0034822.s001.pdf]
