# Supplementary material for: Molecular Etiology of Atherogenesis – In Vitro Induction of Lipidosis in Macrophages with a New LDL Model
Source: PLoS One. 2012 Apr 13;7(4):e34822. doi: 10.1371/journal.pone.0034822 (PMC3325953; doi:10.1371/journal.pone.0034822)
Supplement: Methods S1 — Synthesis of 3H-Chs. Lipid extraction and preparative TLC. Intracellular Chs hydrolysis. (PDF) [file pone.0034822.s002.pdf]

Supporting Information: Molecular etiology of atherogenesis – *In vitro* induction of lipidosis in macrophages with a new LDL model

Luis M.B.B. Estronca[1], Joao C.P. Silva[1], Julio L. Sampaio[2], Andrej Shevchenko[2], Paul Verkade[3], Alfin D.N. Vaz[4], Winchil L.C. Vaz[5], and Otilia V. Vieira[1,*]

[1] CNC - Center for Neuroscience and Cell Biology, University of Coimbra, Largo Marquês de Pombal, 3004-517 Coimbra, Portugal.

[2] Max-Planck Institute for Molecular Cell Biology and Genetics. Pfotenhauerstrasse 108, 01307 Dresden, Germany.

[3] Schools of Biochemistry, and Physiology and Pharmacology, Medical Sciences, University of Bristol, University Walk, Bristol BS8 1TD, United Kingdom.

[4] Pharmacokinetics, Dynamics & Metabolism, Pfizer Global Research and Development, Groton, CT 06340, U.S.A.

[5] Department of Chemistry, University of Coimbra, 3004-535 Coimbra, Portugal.

* E-mail: vieira@cnc.cj.uc.pt

Methods S1

Synthesis of ^{3}H-Chs

Cholesterol (100mg) was mixed with 1mCi of ^{3}H-cholesterol (43.0 Ci/mmol) in 1ml toluene that was evaporated and then 40 mg of succinic anhydride (40 mg/ml in pyridine) was added. The reaction was allowed to occur for 24h at 37ºC with agitation. The solvent was then evaporated and the residue dissolved in 1ml chloroform:methanol (4:1). ^{3}H-Chs was identified by TLC using chloroform:methanol:ammonia (80:20:1, v/v) as eluent using commercially available Chs as a standard and purified by preparative TLC using the same eluent. The purified ^{3}H-Chs was dissolved in 3ml choloform:methanol (4:1) and stored at -20ºC.

Lipid extraction and preparative TLC

After incubation with Nat-LDL, Ac-LDL, Chs-LDL or Chs-POPC liposomes, macrophages were harvested by scraping into 1 ml of distilled water. The cellular lipids were extracted as described elsewhere [1]. Briefly, lipids were extracted with chloroform-methanol (2:1, v/v) and dried by evaporation. The extracted lipids analyzed by TLC on silica gel plates (2 mm thickness, Merck, Darmstadt, Germany) using

hexane:diethyl ether:acetic acid (90:10:1, v/v/v) as eluent. The TLC plates were then immersed for 1 s in a manganese (II) chloride-sulfuric acid reagent (0.2g manganese chloride tetrahydrate is dissolved in 30ml water, to which 30ml methanol is added; 2ml concentrated sulfuric acid is carefully added drop wise), and then heated to 120ºC for 15min. Lipid bands were visualized under long-wavelength UV light (λ=365nm).

Intracellular Chs hydrolysis

Raw cells were incubated with radioactive ^{3}H-Chs-POPC liposomes, at a dose corresponding to a treatment with 300 µg/ml of Chs-LDL (1000/1 molar ratio), for 24 and 48 h at 37ºC. After incubation, the cells were rinsed with DMEM with 0.5% BSA at pH 3.4 for 10 min on ice, then with PBS, and finally with distilled water. Macrophages were scraped off into 1 ml distilled water and 50 µl of this cell suspension was used to quantify the cell protein. Lipids were extracted from the rest of the cell suspension as described in the legend of the Figure S1. The extracted lipids were separated by TLC on silica gel plates (2 mm thickness, Merck, Darmstadt, Germany) using chloroform:methanol (4:1, v/v) with 2% ammonia (pH~10) as eluent. Lipid bands corresponding to Chs, free cholesterol, and cholesteryl esters were scraped and put directly in vials with scintillation liquid and the radioactivity was measured.

References

1. Folch J, Lees M, Sloane Stanley GH (1957) A simple method for the isolation and purification of total lipides from animal tissues. J Biol Chem 226:497-509.
