# Supplementary material for: Molecular Etiology of Atherogenesis – In Vitro Induction of Lipidosis in Macrophages with a New LDL Model
Source: PLoS One. 2012 Apr 13;7(4):e34822. doi: 10.1371/journal.pone.0034822 (PMC3325953; doi:10.1371/journal.pone.0034822)
Supplement: Table S1 — Hydrolysis and re-esterification of cholesterol derived from 3H-Chs in RAW cells. RAW cells were treated for 24 and 48 h with 3H-Chs-POPC liposomes (45∶55). The compounds were separated by TLC prior to measurement of radioactivity. (PDF) [file pone.0034822.s003.pdf]

Table S1: Hydrolysis and re-esterification of cholesterol derived from ^{3}H-Chs in RAW cells.

Radioactive Compound	% of Total Radioactivity	
	24 h	48 h
Chs	92.6 ± 7.4	90.6 ± 11.5
Free Cholesterol	5.6 ± 1.1	7.4 ± 1.1
Cholesteryl Esters	1.8 ± 0.4	2.0 ± 0.8

RAW cells were treated for 24 and 48h with ^{3}H-Chs-POPC liposomes (45:55). The compounds were separated by TLC prior to measurement of radioactivity.
